# Cellular senescence changes and their association with brain structure in schizophrenia and major depressive disorder

**DOI:** 10.21203/rs.3.rs-8177826/v1

**Published:** 2026-10-14

**Authors:** Naohiro Okada, Johanna Seitz-Holland, Tempei Ikegame, Favour Omileke, Masahiro Kiyota, Eimu Shoji, Shunsuke Mori, Susumu Morita, Daisuke Koshiyama, Yoshihiro Satomura, Shinsuke Koike, SEIICHIRO JINDE, Sho Yagishita, Kouhei Kamiya, Osamu Abe, Martha Shenton, Marek Kubicki, Kiyoto Kasai

## Abstract

Individuals with schizophrenia (SZ) and major depressive disorder (MDD) may experience accelerated biological aging, potentially linked to cellular senescence and the senescence-associated secretory phenotype (SASP). While the SASP index has been reported to be elevated in MDD, its status in SZ and its relationship with brain structure remain unclear. This study investigated whether the SASP index, a composite biomarker-based measure, is elevated in SZ and MDD, and examined its associations with brain-predicted age difference (brain-PAD) and regional brain morphology across SZ, MDD, and healthy controls (HCs). A total of 119 subjects (29 individuals with SZ, 40 individuals with MDD, and 50 HCs) underwent both peripheral blood collection and MRI scanning. First, across all subjects, the effects of diagnostic groups on SASP indices were assessed. Second, associations between age-adjusted SASP indices and brain-PAD were examined within each diagnostic group. Third, exploratory analyses tested associations between SASP indices and subcortical volumes as well as cortical thickness and surface area in each diagnostic group. We found a significant main effect of diagnostic group on SASP indices and significantly elevated SASP indices in both SZ and MDD. Although no significant associations were observed between SASP indices and brain-PAD in any diagnostic group, negative associations were identified in SZ between SASP indices and the surface area of the left transverse temporal gyrus and the right inferior frontal gyrus pars triangularis, regions that play pivotal roles in auditory and language processing alterations observed in SZ. These findings suggest that SASP-related processes may contribute to the neural underpinnings of SZ through localized, rather than global, alterations in brain structure.

## INTRODUCTION

Individuals with schizophrenia (SZ) have a life expectancy shortened by approximately 15 years compared to the general population,[1] possibly partly due to an increased prevalence of aging-related systemic diseases, including cardiovascular, infectious, respiratory, and neoplastic diseases.[2, 3] These physical morbidities are attributed to unhealthy lifestyle behaviors, such as substance abuse and physical inactivity.[3] Depression is associated with cardiovascular diseases,[4] and the estimated life expectancy of individuals with depression is more than 5 years shorter than that of controls.[5] In addition to shortened lifespan driven by physical comorbidities, individuals with severe psychiatric disorders may also experience premature decline in cognitive functions, suggesting that accelerated aging may manifest both systemically and neurologically. In individuals with psychotic disorders including SZ, cognitive functions decline more rapidly than would be expected from normal aging.[6] Similarly, individuals with depression exhibit moderate cognitive deficits across a range of domains.[7] Thus, it is suggested that accelerated biological aging may be associated with severe psychiatric disorders including SZ and depression.[8] This hypothesis is supported by evidence that psychological factors are robust predictors of healthspan and longevity.[9] However, it is still unknown whether accelerated aging contributes to the onset and/or progression of psychiatric disorders or whether psychiatric disorders themselves accelerate biological aging.

Accelerated systemic aging may be driven by processes that begin at the cellular level, progressing through tissue and organ-level changes.[10] In this context, growing attention has been paid to the possible association between cellular senescence, which refers to irreversible cell-cycle arrest,[11] and the neurophysiology of psychiatric disorders including SZ. Cellular senescence is a physiological process that plays important roles in development and tissue homeostasis. However, during aging or under certain pathological conditions, senescent cells gradually accumulate and can negatively impact the whole body. These effects are largely mediated by the senescence-associated secretory phenotype (SASP), through which senescent cells release inflammatory cytokines, chemokines, growth factors, angiogenic factors, and matrix metalloproteinases (MMPs).[12] Chronic systemic inflammation, vascular changes, protein remodeling, and repeated cycles of cell proliferation can all promote cellular senescence. In turn, senescent cells exacerbate inflammation and tissue remodeling, creating a self-perpetuating cycle that accelerates organ aging. Chronic inflammation also accelerates the senescence of immune cells, weakening immune function and reducing the clearance of senescent cells.[13] Several studies have reported that cellular senescence in the body may be related to cognitive decline as well as psychiatric symptoms.[14, 15] Additionally, previous papers have shown a strong relationship between metabolic health and cellular senescence/SASP,[16] and psychiatric disorders including SZ and depression are associated with metabolic abnormalities.[3, 4] Taken together, cellular senescence and SASP may serve as key factors for understanding and identifying accelerated aging across psychiatric disorders and throughout the lifespan.

Considering the multiple interrelated biological mechanisms of SASP, investigating SASP-related proteins as one biomarker-composite index may be more integrative than investigating each individual factor separately for the exploration of the association with clinical characteristics.[17] In this context, in recent years, the association between an integrative index comprising a set of SASP-related proteins (SASP index) and major depressive disorder (MDD) has been investigated, revealing that SASP index is elevated in individuals with this disorder.[18–20] Although there has been no study reporting the altered SASP index in the SZ spectrum disorders, single markers related to SASP have been examined. Meta-analytic studies reported higher levels of interleukin (IL)-6, IL-8, and tumor necrosis factor (TNF)-α in individuals with chronic SZ, first-episode SZ, and acute SZ-spectrum disorders.[21–24] In both acute SZ and drug-naïve individuals with first-episode SZ, pharmacological treatment led to reductions in IL-6 levels.[22, 25] Hayes *et al*. reported higher levels of IL-8 in clinical high-risk (CHR) individuals.[26] Baumeister *et al*. reported that childhood trauma—known to increase the risk of psychiatric disorders including SZ—is associated with higher peripheral levels of IL-6 and TNF-α in adults.[27]

Accelerated cognitive decline is associated with brain atrophy, including reduced hippocampal volume. Neuroimaging studies have reported smaller-than-normal hippocampal size in individuals with SZ and depression,[28, 29] supporting the hypothesis of accelerated aging in these disorders. Another recent large-scale study revealed that, on average, individuals with SZ showed a higher-than-normal brain-predicted age difference (brain-PAD), calculated as predicted brain age minus chronological age, with an average increase of + 3.55 years.[30] These findings suggest that structural brain changes may reflect accelerated aging, but the underlying biological mechanisms remain largely unclear. One potential contributor is cellular senescence and SASP. Few studies have examined the relationship between the SASP and brain structure in psychiatric disorders. Mendes-Silva *et al*. reported an association between higher SASP index and white matter microstructural damage, as indicated by higher mean diffusivity, in the cingulate bundle in late-life MDD.[31] Individual SASP-related proteins have been shown to correlate with brain volume. For example, in both individuals with SZ and healthy controls (HCs), higher blood IL-6 levels have been associated with smaller hippocampal volumes.[32] Another study demonstrated that a cluster characterized by elevated IL-6 and IL-8 levels showed large and widespread volumetric reductions, including in the anterior cingulate.[33] Moreover, Klaus *et al*. found a relationship in individuals with SZ between higher TNF-α levels and higher brain-PAD.[34] However, to the best of our knowledge, no study has yet investigated associations between the SASP index and regional brain macrostructure in SZ or MDD.

Here, we hypothesized that the SASP index is elevated not only in MDD but also in SZ. Based on previous evidence,[18–20, 31] we further hypothesized that the SASP index would be associated with brain structural measures in MDD. Because no previous study has examined the relationship between the SASP index and brain structure in SZ, analyses of this association in SZ were considered exploratory. To test these hypotheses, in the current study, we sought to explore the SASP in a transdiagnostic sample across individuals with SZ, MDD, and HCs. Next, we aimed to study the association between the SASP and brain age in the transdiagnostic sample and in the diagnostic subgroups. In additional exploratory analyses, we investigated the association between the SASP and measures of volume, cortical thickness, and surface area of individual brain regions.

### MATERIALS AND METHODS

## Participants

This study was approved by the Research Ethics Committee, Graduate School of Medicine, the University of Tokyo (Approval No. 3150 and 2094). All methods were performed in accordance with the relevant guidelines and regulations. Written informed consent was obtained from all participants. For participants who were legally minors, informed assent was obtained from the participants and written informed consent was obtained from their primary caregivers. Experienced psychiatrists or psychologists administered clinical assessments. HCs were recruited from the Department of Neuropsychiatry, the University of Tokyo Hospital. Individuals with SZ and MDD were recruited from the in- and outpatient clinics at the Department of Neuropsychiatry, the University of Tokyo Hospital. The exclusion criteria for HCs and individuals included (1) previous history of brain injury with loss of consciousness for five minutes or more, (2) past or present alcohol abuse or dependence, and (3) past or present substance abuse or dependence. HCs were confirmed to have no current or past psychiatric disorders. This screening procedure for HCs was the same as in our previous study.[35] Individuals with SZ

and MDD were required to meet the criteria outlined in the Diagnostic and Statistical Manual of Mental Disorders, Fourth Edition (DSM-IV). The exclusion criteria for individuals included a previous history of electroconvulsive therapy (ECT), because previous MRI studies have reported that ECT induces structural brain changes, which may confound structural MRI findings.[36] A total of 127 subjects (30 individuals with SZ, 41 individuals with MDD, and 56 HCs; mean age: 36.8 years) underwent blood sampling and MRI scanning as described below. Daily doses of antipsychotics (chlorpromazine equivalent) at the day of MRI scanning were recorded for individuals with SZ and MDD. Illness severity was assessed using the Overall version of the Brief Psychiatric Rating Scale (BPRS),[37] which includes five factors (affect, positive symptoms, negative symptoms, resistance, and activation) according to Shafer's study,[38] in individuals with SZ, as well as using the 17-item version of GRID-Hamilton Rating Scale for Depression (GRID-HAMD17)[39, 40] in those with MDD.

### Blood sample collection and analysis

Peripheral venous blood samples were collected in tubes containing anticoagulant (ethylenediaminetetraacetic acid) by experienced medical doctors and centrifuged (3,000 rpm, 10min, 4℃) within 30 min after collection. After separation, plasma was stored at -80°C until sample shipment as described below. The conditions at the time of collection were standardized by asking participants not to eat or drink anything other than water for at least 3 hours after the last meal and by fixing the blood collection time from 1pm to 3pm.

Plasma samples were sent to Myriad RBM® laboratory (Austin, TX, USA, https://rbm.q2labsolutions.com) for quantification of concentrations of SASP-related proteins. A validated Luminex-based multiplex immunoassay (Custom Human Map®) was used for determination of protein levels. While the best way to measure senescence processes is still under discussion, the importance of analyzing the overall effects of senescence, rather than the levels of individual proteins, has recently been emphasized to gain a deeper understanding of the dynamics of senescence processes.[41] In this context, we sought to investigate all the 22 protein markers (markers related to immune-inflammatory control, growth factors, cell surface molecules, and survival factors) that Diniz *et al*. had examined in their SASP analysis,[18] but one of the 22 (insulin-like growth factor binding protein 6) could not be measured due to the limited service available from Myriad RBM®. Thus, plasma levels of the remaining 21 proteins were obtained.

Quantified proteins with more than 30% of values out of the quantitation range were excluded from the following analysis, as performed in another study.[20] Fifty percent of the lower limit of quantitation (LOQ) was assigned to values of less than lower LOQ, and 150 percent of the higher LOQ was assigned to values of more than higher LOQ. SASP-related protein levels were log2 transformed and standardized to z-scores before statistical analysis.

A principal component analysis (PCA) was performed on the levels of all the included SASP-related proteins. Individual weights for each protein were extracted based on the first principal component. A SASP index (mean = 0, standard deviation = 1) was calculated for each participant using the following regression equation: SASP index = $\Sigma_{i=1..n} \beta_i x_i + c$, where n is the number of included SASP-related proteins, $x_i$ represents the individual protein level, and $\beta_i$ denotes the protein's weight.

### Image acquisition and preprocessing

T1-weighted structural MRI data were acquired using a Discovery MR750w 3.0T (GE Healthcare) with a spoiled gradient-recalled sequence and a 24-channel head coil. The following parameters were used: repetition time = 7.7 ms, echo time = 3.1 ms, voxel size = 1.016 × 1.016 × 1.2 $mm^3$, flip angle = 11°, field of view = 260 × 260 × 240 $mm^3$, matrix = 256 × 256, and 200 slices. All T1-weighted images were visually checked to exclude the existence of low signal-to-noise ratios, artifacts, or gross anatomical abnormalities that could influence image preprocessing.

T1-weighted images were preprocessed using the *brainageR* package to calculate a brain-predicted age (https://github.com/james-cole/brainageR/).[42–45] The package takes raw T1-weighted MRI scans and uses SPM12 (https://www.fil.ion.ucl.ac.uk/spm/software/spm12/) for segmentation and normalization. A slightly customized version of FSL's *slicesdir* then generates slice images for quality control. Next, the normalized images are loaded into R via the *RNifti* package, vectorized, and combined after masking the gray matter, white matter, and cerebrospinal fluid vectors. Finally, PCA is performed with R's *prcomp*, retaining the 435 components that explain 80% of the variance, which are then fed into a Gaussian process regression model implemented in *kernlab* to predict brain age. The regression model implemented in *brainageR* was trained on a large independent dataset of 3,377 healthy individuals (mean age: 40.6 years) from publicly available neuroimaging cohorts, which appear comparable to our sample. Importantly, brain-PAD derived from *brainageR* is not equivalent to conventional cortical morphometric measures such as cortical thickness or surface area. Although brain-PAD is expected to partially reflect cortical morphological changes associated with aging, *brainageR* estimates brain age from voxel-wise patterns of whole-brain tissue-segmented T1-weighted MRI images using a Gaussian process regression model, rather than from predefined regional morphometric features.[44, 45] Therefore, brain-PAD represents an integrated measure of brain structural aging that partially overlaps with, but is not identical to, cortical thickness or surface area measurements.

Additionally, T1-weighted images were preprocessed using FreeSurfer software version 5.3 (http://surfer.nmr.mgh.harvard.edu/), as performed in our previous studies.[29, 46] FreeSurfer software automatically provides macrostructure models of the human brain given any adequate T1-weighted structural image, thereby calculating intracranial volume and subcortical regional volumes (16 regions according to the Aseg atlas, **Supplementary Table S1**) as well as cortical anatomical measures including cortical thickness and surface area (68 regions according to the Desikan–Killiany atlas, **Supplementary Table S2**). Cortical thickness and surface area are the two constituents of cortical volume in each region, while regional volume is a product of cortical thickness and surface area. Thus, in this study, cortical thickness and surface area were regarded as cortical measures of interest. Subsequently, all segmentation images were visually inspected to exclude poorly preprocessed data. A total of eight subjects (one individual with SZ, one individual with MDD, and six HCs) were excluded.

## Statistical analysis

All statistical analyses, except where noted, were performed using SPSS 27 (IBM Corp., Armonk, NY). All statistical analyses were two-tailed with a significance threshold of $p < 0.05$. The assumptions for each statistical test were verified before analysis. Prior to the main analysis, the demographic characteristics of each diagnostic group were examined using a one-way analysis of variance (ANOVA) test for continuous data and using a $\chi^2$ analysis test for categorical data.

The overview of our analyses is summarized in Fig. 1. First, in all subjects including SZ, MDD, and HCs, the effect of diagnostic group on SASP indices was assessed using a one-way analysis of covariance (ANCOVA), with age and sex as nuisance covariates. A post-hoc Fisher's least significant difference (LSD) test was also performed to investigate pairwise differences. Additionally, the effect of diagnostic group on brain-PAD was assessed using a one-way ANCOVA, with sex as a nuisance covariate, followed by a post-hoc Fisher's LSD test. Age was not included as a nuisance covariate in this model, because brain-PAD was already adjusted for age.

Second, it was investigated whether age-adjusted SASP indices were associated with brain-PAD using a multiple regression model with sex as a nuisance covariate. Age was not included as a nuisance covariate in this model, because both SASP indices and brain-PAD were already adjusted for age. This analysis was conducted separately in individuals with SZ and MDD, with HCs included for reference. Since the analyses in MDD and SZ served different inferential purposes (i.e., testing the a priori hypothesis that the SASP index is associated with brain-PAD in MDD and exploring this association in SZ) and were conducted in independent diagnostic populations, each diagnostic group was treated as an independent family of statistical tests, and multiple comparison correction was not applied across the two groups. For individuals with SZ and MDD, daily doses of antipsychotics (in chlorpromazine equivalents) were included as an additional nuisance covariate to control for medication effects. Further models were tested by including years of education as an additional covariate, again within each diagnostic group. Additionally, in an exploratory manner, if a significant association was found between age-adjusted SASP indices and brain-PAD, it was then investigated whether brain-PAD was correlated with individual age-adjusted SASP-related proteins using the above model. Next, to investigate diagnosis-common and diagnosis-distinct patterns, we fitted a general linear model to the full sample, with diagnostic group and age-adjusted SASP index (with and without their interaction) as independent variables predicting brain-PAD, while adjusting for sex. Finally, in an exploratory manner, if a significant main or interaction effect of the SASP index on brain-PAD was found, additional analyses were conducted to test whether diagnostic group and individual age-adjusted SASP-related proteins had a significant effect on brain-PAD using the same model.

Third, in additional analyses, it was investigated whether SASP indices were associated with subcortical volumes as well as cortical thickness and surface area. Multiple regression models were used, with age and sex included as nuisance covariates in all analyses, and intracranial volume additionally included for the analyses of subcortical volumes. These analyses were performed on individuals with SZ and MDD, with HCs included for reference. For the SZ and MDD groups, separate models additionally included daily antipsychotic dose (chlorpromazine equivalent) as a nuisance covariate. Further models were tested by including years of education as an additional covariate, again within each diagnostic group. Multiple comparison corrections were performed using R's *p.adjust* package according to the Benjamini-Hochberg false discovery rate (FDR) method, applied separately within each category: the 16 subcortical volumes, the 68 cortical thickness measures, and the 68 cortical surface area measures. Additionally, in an exploratory manner, if a significant association was found, exploratory analyses were then conducted to investigate whether anatomical measures were correlated with the levels of individual SASP-related proteins in the corresponding significant regions using the above model. Next, across all subjects, a general linear model was fitted with diagnostic group and SASP index (with and without their interaction) as independent variables, predicting anatomical measures (only those that showed a significant association in individuals with SZ or MDD), adjusting for age and sex. Additionally, in an exploratory manner, if a significant effect of SASP index or an interaction effect was found on anatomical measures, exploratory analyses were then conducted to examine whether diagnostic group and individual age-adjusted SASP-related proteins had significant effect on the measures using the above model.

An a priori power analysis was conducted using G*Power version 3.1.9.6[47] to determine the required sample size for the primary hypotheses concerning the associations of the SASP index with brain-PAD and structural brain measures. Based on a previous study reporting moderate-to-large associations between the SASP index and DTI measures in late-life depression,[31] an effect size of $f^2 = 0.30$ was assumed a priori. With a two-sided alpha level of 0.05 and a statistical power of 0.80, the required sample size was estimated to be 29 participants.

## RESULTS

A total of 119 subjects (29 individuals with SZ, 40 individuals with MDD, and 50 HCs) were included in the current analysis. The acquisition interval between blood samples and MRI data was a median of 0 days (range 0 to 50 days). In 7 of the analyzed 21 SASP-related proteins, more than 30% of values were out of the quantitation range. Thus, only 14 proteins were used for the calculation of SASP indices. **Table 1** shows the percentage of values out of the quantitation range in each protein and respective weights of proteins included in the statistical analysis. Basic characteristics are demonstrated in **Table 2**. Age and sex were significantly different among diagnostic groups.

First, an ANCOVA with age and sex as nuisance covariates revealed a significant effect of diagnostic group on SASP indices ($F = 3.6$, $p = 0.032$). Post-hoc tests revealed higher SASP indices in SZ ($p = 0.045$) and MDD ($p = 0.018$) compared to HCs, and no significant differences in SASP indices between SZ and MDD ($p = 0.95$) **(**Fig. 2a**)**. Another ANCOVA with sex as a nuisance covariate showed a significant effect of diagnostic group on brain-PAD ($F = 9.9$, $p = 1.1 \times 10^{-4}$). Post-hoc tests revealed higher brain-PAD in SZ compared to HCs ($p = 1.7 \times 10^{-4}$) and MDD ($p = 9.6 \times 10^{-5}$), and no significant differences in brain-PAD between HCs and MDD ($p = 0.80$) **(**Fig. 2b**)**.

Second, multiple regression analyses controlling for sex showed that age-adjusted SASP indices were not significantly associated with brain-PAD in any diagnostic group (SZ: $p = 0.17$, MDD: $p = 0.86$, HCs: $p = 0.50$). After additionally controlling for daily doses of antipsychotics, SASP indices remained not significantly associated with brain-PAD in individuals with SZ ($p = 0.17$) or in those with MDD ($p = 0.69$). After controlling additionally for years of education, age-adjusted SASP indices were still not significantly associated with brain-PAD in any diagnostic group (SZ: $p = 0.19$, MDD: $p = 0.69$, HCs: $p = 0.52$). Next, the effect of the diagnostic group and age-adjusted SASP index on brain-PAD was examined, and the diagnostic group ($F = 9.5$, $p = 1.5 \times 10^{-4}$) but not age-adjusted SASP index ($F = 0.50$, $p = 0.48$) had significant effects. The interaction effect between diagnostic group and age-adjusted SASP indices on brain-PAD was also investigated in another model, which was not significant ($F = 1.3$, $p = 0.28$).

Third, multiple regression analyses controlling for age and sex showed that SASP indices were significantly negatively associated with the surface area of the left transverse temporal gyrus (TTG) (FDR-corrected $p = 0.039$, uncorrected $p = 6.4 \times 10^{-4}$) and right inferior frontal gyrus pars triangularis (IFG-PT) (FDR-corrected $p = 0.039$, uncorrected $p = 1.2 \times 10^{-3}$) in individuals with SZ **(**Fig. 3a**)**, whereas no significant results were found in all subjects or in individuals with MDD or in HCs. After controlling also for daily doses of antipsychotics, SASP indices remained significantly negatively associated with the surface area of the left TTG (FDR-corrected $p = 0.042$, uncorrected $p = 1.2 \times 10^{-3}$) and right IFG-PT (FDR-corrected $p = 0.042$, uncorrected $p = 1.2 \times 10^{-3}$) in individuals with SZ. After controlling for years of education, SASP indices remained significantly negatively associated with the surface area of the left TTG (FDR-corrected $p = 0.034$, uncorrected $p = 9.6 \times 10^{-4}$) and right IFG-PT (FDR-corrected $p = 0.034$, uncorrected $p = 1.0 \times 10^{-3}$) in individuals with SZ, while no significant results were found in those with MDD or in HCs. Based on the reported association between positive symptoms and the left TTG in individuals with SZ[48–52], an exploratory subgroup analysis was conducted according to positive symptom severity assessed using the BPRS positive symptom factor score (median score: 9). The association between the SASP index and the surface area of the left TTG was significantly negative in the higher positive symptom group (BPRS positive symptom factor score ≥ 10; $p = 0.038$), whereas this association was not significant in the lower positive symptom group (BPRS positive symptom factor score ≤ 9; $p = 0.15$). Some SASP-related proteins such as gp130, tissue inhibitor of metalloproteinases-1, and tumor necrosis factor receptor-2 were significantly negatively associated with the surface area of the left TTG and/or right IFG-PT in individuals with SZ **(Supplementary Table S3)**. Next, the effect of the diagnostic group and SASP index on the surface area of the left TTG and right IFG-PT was examined, and the SASP index ($F = 8.3$, $p = 4.8 \times 10^{-3}$ [left TTG]; $F = 4.9$, $p = 0.030$ [right IFG-PT]) but not the diagnostic group ($F = 1.8$, $p = 0.17$ [left TTG]; $F = 0.59$, $p = 0.56$ [right IFG-PT]) had significant effects. The effects of the interaction between diagnostic group and SASP index on the surface area of these two measures were also investigated in another model, while the interaction effects were trend-level significant ($F = 2.5$,

$p$ = 0.089) for the left TTG and significant ($F$ = 4.3, $p$ = 0.015) for the right IFG-PT (Fig. 3). Associations of SASP indices with cortical surface area of the left TTG and right IFG-PT in individuals with MDD and HCs are displayed in Fig. 3b-c. Effects of some individual SASP-related proteins or their interaction effects with diagnostic group were significant on the surface area of the left TTG and/or right IFG-PT in all subjects **(Supplementary Table S4)**.

## DISCUSSION

In this study, we found a significant main effect of diagnostic group on SASP indices and significantly higher-than-normal SASP indices in SZ and MDD. Furthermore, although there were no significant associations between SASP indices and brain-PAD within any diagnostic group, we identified negative associations between SASP indices and the surface area of the left TTG and the right IFG-PT, exclusively in SZ.

We found significantly higher SASP indices in individuals with SZ than in HCs (Fig. 2a). To the best of our knowledge, this is the first study to demonstrate a higher overall SASP index in SZ, integrating multiple SASP-related proteins that have previously been reported as altered in SZ spectrum disorders.[21, 23–25, 53] These findings are consistent with the hypothesis that individuals with SZ may experience aspects of premature or accelerated aging. We also found significantly higher SASP indices in individuals with MDD compared to HCs (Fig. 2a). Cellular senescence has been suggested to contribute to autonomic nervous system dysfunction and depressive symptoms.[54, 55] Several previous studies reported higher SASP indices in adults with late-life MDD[18] and young and middle-aged adults experiencing a current major depressive episode.[20] Meta-analyses have further shown associations between elevated levels of inflammatory proteins and poorer treatment response in MDD, and a recent study revealed that higher SASP indices were linked to higher rates of non-remission in older adults with MDD.[17] Our results replicate and extend these previous findings. Notably, our results suggest that SASP indices may serve as a key transdiagnostic biomarker across SZ and MDD.

We found no significant association between SASP indices and brain-PAD in any diagnostic group. This lack of a significant association is not in line with our hypothesis. We also found that brain-PAD was elevated only in SZ (Fig. 2b), despite the general tendency for increased brain-PAD across neuropsychiatric conditions.[56] Given the elevated SASP indices in both SZ and MDD (Fig. 2a), SASP and brain aging may each contribute to the etiology of SZ, possibly in partly independent ways. We suggest that the SASP is a peripheral marker for cellular senescence which is increased in both disorders, while brain-PAD is a central marker for "aging" (even though this can be debated) which seems to be affected only in SZ, and additionally they do not seem to be related to each other. Furthermore, SASP indices and brain age show differential patterns across the three diagnostic groups, which may eventually help distinguish them. Future studies will be required to validate these hypotheses and elucidate the underlying mechanisms.

We found negative associations between SASP indices and the surface area of the left TTG and the right IFG-PT only in SZ (Fig. 3). Given the absence of significant association between SASP indices and brain-PAD in all subjects or in any diagnostic group, the relationship between SASP expression and brain structure may be region-specific rather than global. According to previous studies, both the superior temporal gyrus, part of which is the TTG, and the inferior frontal gyrus are among the most likely affected brain regions in SZ.[57] Several studies have reported smaller volumes of the left TTG, or Heschl's gyrus in SZ.[50–52] Individuals with first-episode SZ showed significant decreases in gray matter volume over time in the left TTG.[48] A recent large-scale study revealed smaller surface area in the left TTG (Cohen's $d$ = – 0.15) in individuals with SZ compared to HCs.[58] The superior temporal gyrus, including the TTG, is known for its role in auditory processing. Smaller volume in the left TTG has been associated with hallucinations and delusions in first-episode psychosis.[59] Thus it would be suggested that, in SZ, cellular senescence may be partly related to smaller left TTG, a region implicated in psychotic symptoms.[59] Previous studies reported smaller volumes of the right IFG-PT in SZ[60] and even in individuals with first-episode SZ and ultra-high risk for psychosis.[61] A recent large-scale study revealed smaller surface area in the right IFG-PT (Cohen's $d$ = – 0.14) in individuals with SZ compared to HCs.[58] The IFG-PT, as part of Broca's area, plays a role in language processing. Volumes of the right IFG-PT are smaller in the cognitively deteriorated group compared to healthy subjects and cognitively preserved groups with SZ.[62] Moreover, smaller volume of the right IFG-PT is associated with poor emotion recognition,[63] and disorganized and positive symptoms[64] in individuals with SZ. In this context, in SZ, cellular senescence may be related to smaller right IFG-PT, which is known to be associated with cognitive/disorganized and social symptoms.[62–64] Previous longitudinal studies reported progressively decreased volumes in the left TTG[48, 49] and the right inferior frontal gyrus after the onset of first-episode SZ.[49, 65] Both regions are affected even in CHR individuals,[61, 66] and volumetric reductions are also observed in relatives of individuals with SZ,[67–69] implying the effects of genetic risk factors for SZ on the shrinkage of these brain regions. Subsequently, it is possible that genetic factors may first affect both the left TTG and right IFG-PT, leading to regional vulnerability. Then, SASP-related phenomena, derived from other factors such as systemic diseases and environmental stressors, may cause these regions to shrink during the peri-onset period, in line with the two-hit model of SZ. However, this model remains just a hypothesis, the detailed mechanism of which warrants further investigation in the future.

There are some limitations in this study. First, only 14 SASP-related proteins out of the 22 included in the previous study were used for the calculation of SASP indices mainly due to the exclusion of those with large amounts of values out of the quantitation range. Second, the current study is cross-sectional in nature and the participants were in their chronic phase of SZ; thus, the causal relationships between SASP indices and brain structure and its timing through the life-course for developing psychosis were not determined. Third, although antipsychotic dosage was recorded and adjusted for in the statistical analyses, plasma antipsychotic concentrations were not measured as part of the study protocol. Because antipsychotics may influence SASP-related biological processes, the potential confounding effect of variability in antipsychotic exposure cannot be excluded. Future studies incorporating therapeutic drug monitoring may help clarify whether differences in plasma antipsychotic concentrations contribute to the observed associations. Finally, almost all the subjects of this study were Japanese people. Further investigation is needed to examine if the results of this study can be shared in various ethnicities and races.

In conclusion, we found a significant main effect of diagnostic group on SASP indices and significantly higher-than-normal SASP indices in SZ and MDD. Furthermore, although there were no significant associations between SASP indices and brain-PAD within any diagnostic group, we identified negative associations between SASP indices and the surface area of the left TTG and the right IFG-PT, exclusively in SZ. These findings suggest that SASP-related molecules may contribute to the neural substrates of SZ through their association with regional but not global brain structure.

### DECLARATIONS

## ETHICS DECLARATION

**Conflict of Interest**

All authors declare no competing interests.

## FUNDING

This work was supported by MEXT/JSPS KAKENHI, grant numbers JP18K15478 (N.O.), JP21K15709 (N.O.), JP22H04926 (N.O.), JP21H05171 (K.Kas.), and JP21H05174 (K.Kas.), JST Moonshot R&D, grant number JPMJMS2021 (K.Kas.), and AMED, grant numbers JP18dm0307001 (K.Kas.), JP18dm0307004 (S.K. and K.Kas.), JP19dm0207069 (S.K. and K.Kas.), and JP23wm0625001 (K.Kas.). J.S-H. was supported by the Harvard Medical School Livingston Fellowship Award, the BBRF Young Investigator grant (funded by Mary and John Osterhaus and the Brain & Behavior Research Foundation), a NIMH-funded K99MH131850, and a Pilot Award from the Women's Brain Initiative from the Brigham Research Institute. This work was also partially supported by WPI-IRCN, UTIAS.

## AUTHOR CONTRIBUTIONS

N.O., J.S.-H., M.E.S., M.Ku., and K.Kas. designed the research. N.O., T.I., S.Morit., D.K., Y.S., S.J., K.Kam., and O.A. performed the research. N.O. analyzed the data. N.O., J.S.-H., F.O., M.Ki., E.S., S.Mori, S.K., S.J., S.Y., M.E.S., M.Ku., and K.Kas. wrote the paper.

## ACKNOWLEDGEMENTS

The authors would like to highly thank participants in this study.

### DATA AVAILABILITY

The datasets generated during and/or analyzed during the current study are not publicly available due to ethical reasons but are available from the corresponding author on reasonable request.

## TABLES

Table 1 and 2 are available in the Supplementary Files section.

### Figures

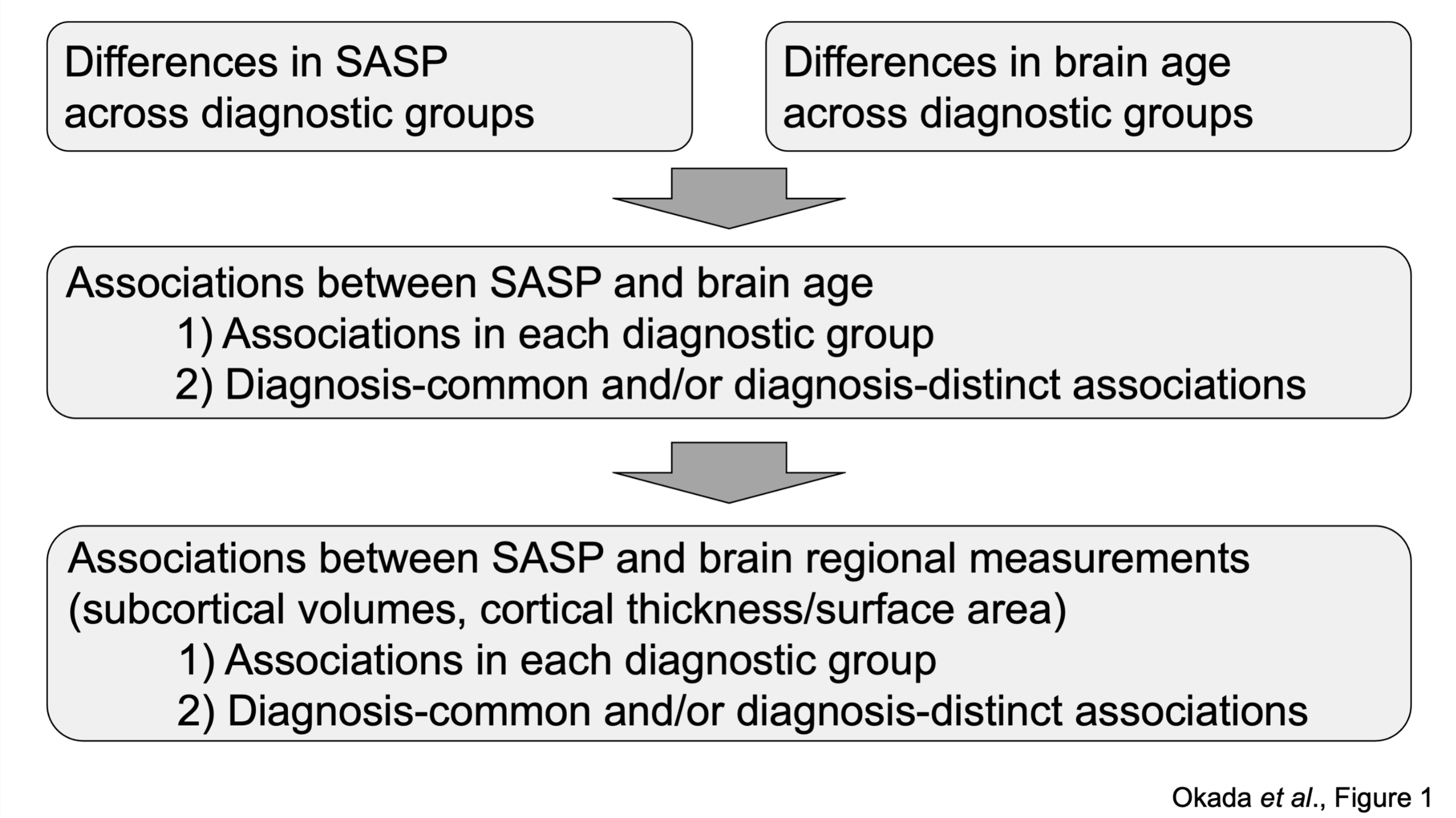

**Figure 1**

Schematic overview of the statistical analyses performed in the current study.

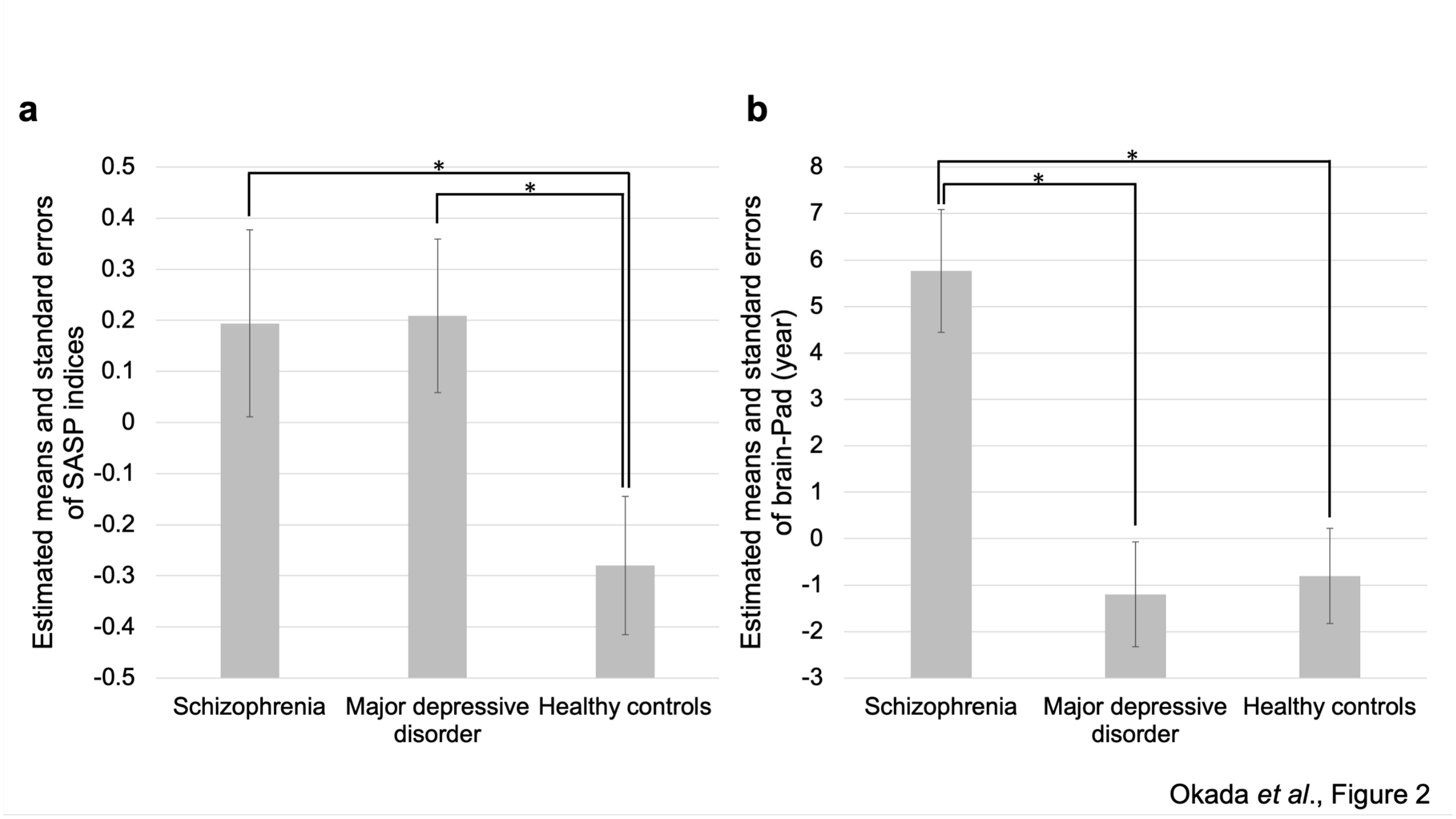

**Figure 2**

Differences in SASP indices and brain age across diagnostic groups. **(a)** Estimated marginal means and standard errors of SASP indices in each diagnostic group, adjusted for age and sex, are shown. **(b)** Estimated marginal means and standard errors of brain-predicted age difference (brain-PAD) in each diagnostic group, adjusted for sex, are shown. * represents $p < 0.05$ in the ANCOVA post-hoc tests.

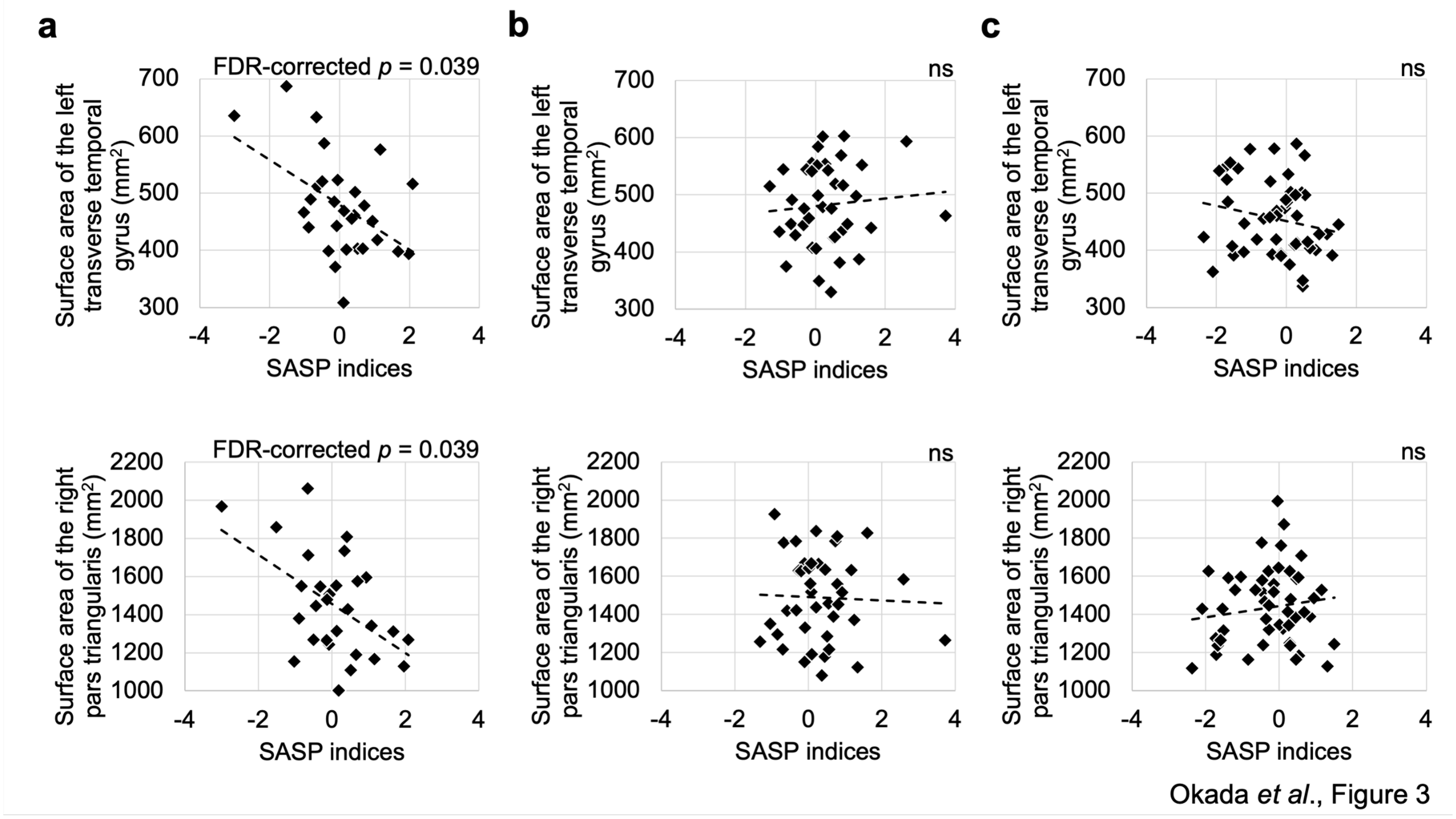

**Figure 3**

Associations between SASP indices and anatomical measures in each diagnostic group (**a**, schizophrenia; **b**, major depressive disorder; **c**, healthy controls). Associations are displayed only for regions where a significant association was found in either schizophrenia or major depressive disorder. Abbreviation: ns, not significant.

## Supplementary Files

This is a list of supplementary files associated with this preprint. Click to download.

- Table1SASPOkada20251120.xlsx
- Table2SASPOkada20260802.xlsx
- SupplementarySASPOkada20251120.pdf
